# Age and Sex Differences of Comorbidities in Medicare Older Adults With Chronic Obstructive Pulmonary Disease

**DOI:** 10.1093/geroni/igaa057.381

**Published:** 2020-12-16

**Authors:** Tham Le, Linda Simoni-Wastila

**Affiliations:** University of Maryland, Baltimore, Maryland, United States

## Abstract

OBJECTIVES: Although comorbidity varies by sex and age, comorbidity variation among individuals with specific primary conditions is less well-understood. We sought to quantify chronic comorbidities in older adults with chronic obstructive pulmonary disease (COPD) using representative Medicare claims data. METHODS: This retrospective cohort study consisted of individuals aged 65+ with a COPD diagnosis identified in a 5% Medicare sample enrolled between 1/1/2012-1/1/2015. We quantified the prevalence of 40 comorbidities and sex and age variation (≥ or ≤ 85 years). RESULTS: Of 461,268 eligible beneficiaries, 60% were female, 86% were white, with mean (SD) age of 79 (8) years. The majority (89.2%) had at least 5 comorbidities; 50.4% had ≥ ten comorbidities. Most prevalent conditions included: hypertension (92.6%), hyperlipidemia (86.4%), anemia (74.9%), rheumatoid arthritis (RA) (68.0%), congestive heart failure (CHF) (49.6%), diabetes (46.7%), depression (43.7%), peripheral vascular disease (PVD) (42.9%), and chronic kidney disease (CKD) (38.6.) Male patients had higher prevalence of CHF, diabetes, CKD, atrial fibrillation (AFib), AMI, cancer, tobacco use disorder, and liver disease, while females had higher prevalence of hypertension, anemia, RA, depression, asthma, osteoporosis, pain, hypothyroid, obesity, dementia, and glaucoma. Compared to patients under 85, those aged ≥85 years had higher prevalence of cardiovascular disease, depression, musculoskeletal conditions, cancer, dementia, glaucoma, and CKD; but lower prevalence of asthma, anxiety, and metabolic disorders. CONCLUSIONS: Older adults with COPD encounter a high prevalence of comorbidities. Comorbidity patterns differs across age and sex spectrum, highlighting the significance of age and sex in developing individualized clinical care and epidemiological research.

